# Assessing health determinants worldwide: Econometric analysis of the Global Burden of Diseases Study 2000–18 – Highlighting impactful factors on DALY, YLL, and YLD indicators

**DOI:** 10.7189/jogh.14.04051

**Published:** 2024-03-15

**Authors:** Kamran Irandoust, Rajabali Daroudi, Maryam Tajvar, Mehdi Yaseri

**Affiliations:** 1 Department of Health Management, Policy, and Economics, School of Public Health, Tehran University of Medical Sciences, Tehran, Iran; 2 Department of Health Economics, School of Health Management and Information Sciences, Iran University of Medical Sciences, Tehran, Iran; 3 Department of Epidemiology and Biostatistics, School of Public Health, Tehran University of Medical Sciences, Tehran, Iran

## Abstract

**Background:**

As the health status of a population is influenced by a variety of health determinants, we sought to assess their impact on health outcomes, both at the global and regional levels.

**Methods:**

This ecological study encompassed all 194 member countries of the World Health Organization (WHO) from 2000 to 2018. We first identified all health determinants and then retrieved the related data from various global databases. We additionally considered three indicators – disability-adjusted life years (DALYs), years of life lost (YLL), and years lived with disability (YLD) – in evaluating health outcomes; we extracted their data from the Global Burden of Disease (GBD) 2019 study. We then applied econometric analyses using a multilevel mixed-effects linear regression model.

**Results:**

The analysis using the DALY indicator showed that the variables of sexually transmitted infections, injuries prevalence, and urbanisation had the highest effect size or regression coefficients (*β*) for health outcomes. The variables of sexually transmitted infection (*β* = 0.75, *P* < 0.001) in the African region; drinking water (*β* = −0.60, *P* < 0.001), alcohol use (*β* = 0.20, *P* < 0.001), and drug use (*β* = 0.05, *P* = 0.036) in the Americas region; urbanisation (*β* = −0.34, *P* < 0.001) in the Eastern Mediterranean region; current health expenditure (*β* = −0.21, *P* < 0.001) in the Europe region; injuries (*β* = 0.65, *P* < 0.001), air pollution (*β* = 0.29, *P* < 0.001), and obesity (*β* = 0.92, *P* < 0.001) in the South-East Asia region; and gross domestic product (*β* = −0.25, *P* < 0.001), education (*β* = −0.90, *P* < 0.001), and smoking (*β* = 0.28, *P* < 0.001) in the Western Pacific region had the most significant role in explaining global health outcomes. Except for the drug use variable in regional findings, the role of other variables in explaining the YLL indicator was greater than that of the YLD indicator.

**Conclusions:**

To address global health disparities and optimise resource allocation, global and interregional policymakers should focus on determinants that had the highest *β* with health outcomes in each region compared to other regions. These determinants likely have a higher marginal health product, and investing in them is likely to be more cost-effective.

Health is one of the most important aspects of human life and has consequently been the focus of many human efforts. Its meaning is broad; it changes over time and depends on the level of awareness and perception in societies with different geographical and cultural conditions. In the past, health was often defined simply as not being sick [1]. However, the World Health Organization (WHO) has more comprehensively defined it as a state of complete physical, mental, and social well-being and not merely the absence of disease or infirmity. According to this definition, we understand that health is a multidimensional issue [2] comprising physical, mental, and social dimensions that are interconnected and influence each other. Physical problems can affect a person's mental health, mental problems can affect the body, and both can impact society. Disorders in society, in turn, affect both physical and mental health. Therefore, measures taken to promote health should address all aspects of health [3,4].

To promote these dimensions, besides medical interventions and clinical care, special attention should be paid to non-medical determinants of health. Each of these determinants, either individually or by influencing each other, strongly affects and can lead to inequalities in health status [5,6]. Although medical care and clinical interventions can contribute to longevity and recovery from serious disease, non-medical factors or health determinants that cause people to get sick or require clinical care are often more important for the health of a population [7]. According to studies, the health care system contributes no more than 25% to promoting health, while other determinants affect the remaining 75% [8,9].

This underscores that factors such as income, education, occupation, nutrition, and social class exert more influence on the occurrence of diseases than biological factors, thus playing a pivotal role in human health. Neglecting these determinants of health can impede efforts towards global health goals, which makes understanding them and their contributions to health disorders highly necessary [10]. For this reason, the WHO has initiated a significant endeavour to investigate social determinants of health and health equity; it established a commission on social determinants of health, comprising experts and scientists tasked with gathering evidence on social factors influencing health and promoting equitable health care [11].

Furthermore, numerous researchers have developed models to examine the role and impact of these determinants on health, exploring their interplay in diverse health conditions and devising strategies to achieve health goals [12]. One such example is the County Health Rankings & Roadmaps (CHR&R) developed by the University of Wisconsin Population Health Institute in collaboration with the Robert Wood Johnson Foundation. This model categorises determinants into four groups – clinical care; social and economic factors; physical environment; and health behaviours [13,14].

Existing studies on the role and impact of these determinants on health and their interplay in various health conditions have primarily focussed on specific countries or national-level effects, while others have concentrated on the influence of specific determinants while overlooking other critical factors. Recognising the limitations of prior research, we aimed to investigate and compare the role of health determinants in health outcome across all WHO member countries, both collectively and individually by region. Unlike most previous studies, we sought to consider not only specific factors but all determinants, encompassing socioeconomic factors, clinical care, physical environment, and health behaviours. Since understanding the impact and role of each determinant on health is vital for informed decision-making and the prioritisation of efficient and effective resource allocation policies, we also wanted to explore the impact of each determinant at regional levels.

## METHODS

This ecological study is a descriptive-analytical investigation comprising all 194 member-countries of the WHO. We extracted data from global databases covering the period from 2000 to 2018 and used econometric analyses to investigate the effect of determinants on health. The study received approval from the ethics committee of the School of Public Health, Tehran University of Medical Sciences (Ref: IR.TUMS.SPH.REC.1398.335). Overall, we conducted this study using an eight-stage process.

### Stage 1: Health determinants model selection

First, we had to identify and select health determinants according to a specific pattern or model. Various models have been designed worldwide to explore health determinants, each with its advantages and disadvantages. The choice of a model depends on a study's requirements, prevailing ideologies, and the correct modelling approach. In our study, given its requirements and ecological nature, we critically examined and compared different models with expert consultation, ultimately opting for the CHR&R model to conduct this research. Widely used for ecological studies, especially in the USA, it was developed through collaboration between the Robert Wood Johnson Foundation and the University of Wisconsin Population Health Institute (Figure S1 in the **Online Supplementary Document**). The model aims to promote health for all and eliminate health disparities; raise awareness of factors influencing health; create a reliable data source; and involve health leaders in creating sustainable changes. It categorises health determinants into 13 subcomponents placed in four components: Clinical care; social and economic factors; physical environment; and health behaviours [15].

### Stage 2: Indicator selection according to the model

In this stage, guided by the model chosen in Stage 1, we explored and looked for indicators to measure the determinants (variables) within the model. To achieve this, we searched global databases from which we had decided to collect data, selecting indicators for each health determinant that could effectively represent that specific determinant. These selections were informed by previous studies and expert opinions. Through this method, we identified 36 primary indicators to assess the 13 determinants of health (Tables S1–2 in the **Online Supplementary Document**). Additionally, we selected three indicators to measure health outcomes – disability-adjusted life years (DALYs), years of life lost (YLL), and years lived with disability (YLD).

### Stage 3: Country identification and selection by region

We included all 194 WHO member-countries, grouping them by region (Table S3 in the **Online Supplementary Document**).

### Stage 4: Data collection for selected indicators and countries

After selecting the indicators and countries, we collected data for all 39 indicators (36 health determinants indicators and 3 health outcome indicators) from the 194 WHO member-countries for the 2000–18 period. To achieve this, we extracted data related to 36 health determinants indicators from various databases, including the World Bank, World Health Observatory, Global Health Expenditure Database, Gapminder, United Nations Human Development Reports, and the Global Burden of Disease (GBD) 2019 study; we retrieved data on the three health outcome indicators from the lattermost of these databases (Table S4 in the **Online Supplementary Document**). If indicators with data were available in more than one database, we used the one containing the most comprehensive data.

### Stage 5: Panel data file creation

Given that the data encompassed both time series (2000–18) and cross-sectional data (all WHO member-countries), we constructed a data panel in Microsoft Excel 2010 (Microsoft Corporation, Redmond WA, USA), incorporating 36 indicators of health determinants and three indicators of health outcome for 194 countries between 2000 and 2018.

### Stage 6: Data refinement

In this stage, we first ensured the accuracy of the data and then proceeded to eliminate any outlier data points so as to prevent the occurrence of spurious regressions in statistical analyses. Consequently, we excluded data related to the population density indicator for Monaco and Singapore. We also calculated the data availability for each of the indicators (able S1 and Table S2 in the **Online Supplementary Document**).

### Stage 7: Finalising indicators

In this stage, we took a series of steps to finalise the study indicators. First, using the multilevel mixed-effects linear regression model, we performed univariate analysis to test the significance of the relationship between the 36 health determinants indicators and the three health outcome indicators. Given the univariate nature of the analysis (Table S5 in the **Online Supplementary Document**), we defined a unique model for each health determinant indicator, resulting in 108 iterations. After conducting this test for all variables, we excluded all indicators with a *P*-value >0.1, followed by those with data availability <80% and any overlapping indicators (Table S2 in the **Online Supplementary Document**). We then performed multiple analyses for the remaining indicators using the multilevel mixed-effects linear regression model, entering all health determinants indicators into the model simultaneously. After this step, we excluded any indicators with a *P*-value >0.1, removing the one with the highest *P*-value >0.1 and then rerunning the model. We repeated this process until no indicator with a *P*-value of 0.1 remained. In this way, indicators such as domestic general government health expenditure for DALYs and YLL and the indicators of unemployment and smoking for YLD had a *P*-value >0.1 and were excluded. After these steps, 13 indicators to explain DALYs, 13 indicators for YLL, and 9 indicators for YLD were entered into the final model for statistical analysis (Table S6 in the **Online Supplementary Document**).

### Stage 8: Statistical model selection and data analysis

In this stage, we initially applied a logarithm transformation to both the health outcome indicators (dependent variables) and the health determinants indicators (independent variables) included in the study. We then employed a multilevel mixed-effects linear regression model to examine the relationship between the independent variables and dependent variable. We set the significance threshold for *P*-values at <0.05. We performed all data analyses in Stata, version 16 (StataCorp LLC, College Station TX, USA) and generated graphical representations in R, version 4.0.3 (R Core Team, Vienna, Austria).

## RESULTS

### Global overview

Our global analytical findings based on the DALY indicator showed that the variables of sexually transmitted infections (regression coefficient (*β*) = 0.43; 95% confidence interval (95% CI)  = 0.37, 0.49, *P* < 0.001), injuries prevalence (*β* = 0.23, 95% CI = 0.18, 0.28, *P* < 0.001), and urbanisation (*β* = −0.21; 95% CI = −0.26, −0.15, *P* < 0.001) played the most significant roles in explaining the global health circumstances. Specifically, a 1% increase in the sexually transmitted infections is associated with a likely increase of 0.43% in DALYs. Similarly, a 1% increase in injuries prevalence is associated with a likely increase of 0.23% in DALYs, and a one-percent increase in urbanisation is associated with a likely decrease of 0.21% in DALYs (**Table 1**).

**Table 1:** Health determinants and their impact on DALYs, YLL, and YLD worldwide (2000–18)

	Linear regression		Log-Log linear regression	
**Variables**	***β* (95% CI)**	***P*-value**	***β* (95% CI)**	***P*-value**
DALYs				
*Current health expenditure*	1.67 (1.04, 2.31)	<0.001	−0.01(−0.02, 0.01)	0.326
*Gross domestic product*	0.08 (0.02, 0.15)	0.012	−0.03 (−0.05, −0.01)	0.003
*Education index*	−21 673.98 (−29640, −13708)	<0.001	−0.19 (−0.23, −0.15)	<0.001
*Unemployment*	127.75 (44.50, 211.00)	0.003	−0.002 (−0.01, 0.01)	0.657
*Injuries prevalence*	0.28 (0.15, 0.41)	<0.001	0.23 (0.18, 0.28)	<0.001
*Urbanisation*	−183.05 (−262.58, −103.53)	<0.001	−0.21 (−0.26, −0.15)	<0.001
*Air pollution*	332.38 (246.58, 418.18)	<0.001	0.03 (0.01, 0.05)	0.015
*Basic drinking-water services*	−200.78 (−265.21, −136.36)	<0.001	−0.15 (−0.19, −0.11)	<0.001
*Alcohol use*	652.58 (355.46, 949.71)	<0.001	−0.003 (−0.01, 0.1)	0.585
*Drug use*	11 107.86 (4056.5, 18159.22)	0.002	0.08 (0.06, 0.11)	<0.001
*Smoking*	−399.65 (−670.50, −128.81)	0.004	−0.07 (−0.11, −0.03)	<0.001
*Prevalence of obesity*	1016.82 (833.75, 1199.88)	<0.001	−0.04 (−0.08, −0.01)	0.019
*Sexually transmitted infections*	1.59 (1.40, 1.77)	<0.001	0.43 (0.37, 0.49)	<0.001
*Year*	−867.57 (−967.11, −768.03)	<0.001	−0.008 (−0.009, −0.007)	<0.001
YLL				
*Current health expenditure*	1.60 (0.97, 2.23)	<0.001	−0.02 (−0.03, 0.00)	0.055
*Gross domestic product*	0.084 (0.02, 0.15)	0.011	−0.04 (−0.07, −0.01)	0.003
*Education index*	−21 232.70 (−29 086, −13 379)	<0.001	−0.16 (−0.21, −0.11)	<0.001
*Unemployment*	129.24 (47.10, 211.39	0.002	−0.01 (−0.02, −0.00)	0.008
*Injuries prevalence*	0.26 (0.13, 0.39)	<0.001	0.34 (0.28, 0.41)	<0.001
*Urbanisation*	−175.39 (−256.13, −100.64)	<0.001	−0.26 (−0.33, −0.19)	<0.001
*Air pollution*	313.60 (229.30, 397.91)	<0.001	0.06 (0.03, 0.09)	<0.001
*Basic drinking-water services*	−198.41 (−261.94, −134.87)	<0.001	−0.10 (−0.15, −0.04)	0.001
*Alcohol use*	622.53 (330.92, 914.15)	<0.001	−0.004 (−0.02, 0.01)	0.503
*Drug use*	10 868.89 (3923.08, 17814.7)	0.002	0.07 (0.03, 0.10)	<0.001
*Smoking*	−396.01 (−662.20, −129.83)	0.004	−0.06 (−0.11, −0.01)	0.021
*Prevalence of obesity*	951.60 (772.46, 1130.74)	<0.001	−0.01 (−0.05, 0.04)	0.782
*Sexually transmitted infections*	1.51 (1.33, 1.69)	<0.001	0.61 (0.53, 0.69)	<0.001
*Year*	−831.60 (−929.07, −734.11)	<0.001	−0.015 (−0.02, −0.01)	<0.001
YLD				
*Current health expenditure*	0.06 (0.04, 0.8)	<0.001	−0.002 (−0.01, 0.00)	0.095
*Education index*	−540.97 (−810.88, −271.06)	<0.001	−0.04 (−0.05, −0.03)	<0.001
*Injuries prevalence*	0.03 (0.03, 0.04)	<0.001	0.10 (0.08, 0.11)	<0.001
*Urbanisation*	−8.71 (−11.98, −5.45)	<0.001	−0.02 (−0.03, −0.01)	0.004
*Air pollution*	8.63 (5.48, 11.78)	<0.001	−0.003 (−0.01, 0.00)	0.252
*Basic drinking-water services*	−4.99 (−7.25, −2.74)	<0.001	−0.03 (−0.04, −0.02)	<0.001
*Drug use*	878.13 (620.25, 1136)	<0.001	0.03 (0.02, 0.04)	<0.001
*Prevalence of obesity*	59.90 (52.85, 66.94)	<0.001	−0.04 (−0.5, −0.03)	<0.001
*Sexually transmitted infections*	0.07 (0.06, 0.08)	<0.001	0.09 (0.07, 0.011)	<0.001
*Year*	−29.77 (−33.53, −26.02)	<0.001	0.001 (0.000, 0.001)	<0.001

*β* – regression coefficient, CI – confidence interval, DALYs – disability-adjusted life years, YLD – years lived with disability, YLL – years of life lost

At the global level, the findings on the YLL and DALY indicators showed that the variables of sexually transmitted infections, injuries prevalence, and urbanisation have the greatest role in explaining YLL. Likewise, the findings related to the YLL indicator also indicate that, at the global level, the variables of injuries prevalence, sexually transmitted infections, and education have the greatest impact on either reducing or increasing the YLD (*P* < 0.05) (**Table 1**).

In this study, we included the year variable in the model to account for temporal effects spanning the years 2000 to 2018. In the global context, we observed a *β* coefficient of −0.008 (95% CI = −0.009, −0.007; *P* < 0.001) for the influence of the year variable on the change in the DALY indicator, indicating a significant negative association. Similarly, we found a* β* coefficient of −0.015 (95% CI = −0.02, −0.01; *P* < 0.001) for the impact of the year variable on the change in the YLL indicator, also demonstrating a significant negative association. Conversely, for the change in the YLD indicator, the *β* coefficient was 0.001 (95% CI = 0.000, 0.002; *P* < 0.001), indicating a small but statistically significant positive association with the year variable. Moreover, the impact of all determinant variables on the change in the YLL indicator was greater than that on the YLD indicator (*P* < 0.05) (**Table 1**).

### Regional insights

Regional analyses based on the DALY indicator showed that the variables of sexually transmitted infection (*β* = 0.75; *P* < 0.001) in the African region; drinking water (*β* = −0.60, *P* < 0.001), alcohol use (*β* = 0.20; *P* < 0.001), and drug use (*β* = 0.05; *P* = 0.036) in the Americas region; urbanisation (*β* = −0.34; *P* < 0.001) in the Eastern Mediterranean region; current health expenditure (*β* = −0.21; *P* < 0.001) in the Europe region; injuries (*β* = 0.65; *P* < 0.001), air pollution (*β* = 0.29; *P* < 0.001), and obesity (*β* = 0.92; *P* < 0.001) in South-East Asia region; and gross domestic product (*β* = −0.25; *P* < 0.001), education (*β* = −0.90; *P* < 0.001), and smoking (*β* = 0.28; *P* < 0.001) in the Western Pacific region had the highest effect size with health outcome (**Table 2**).

**Table 2:** Health determinants and their impact on DALYs, YLL, and YLD across WHO regions (2000–18)

	African		Americas		Eastern Mediterranean		Europe		South-East Asia		Western Pacific	
**Variables**	***β***	***P-*value**	***β***	***P-*value**	***β***	***P-*value**	***β***	***P-*value**	***β***	***P-*value**	***β***	***P-*value**
DALYs												
*Current health expenditure*	−0.03	0.021	−0.04	0.257	−0.12	0.001	−0.21	<0.001	−0.04	0.197	−0.19	<0.001
*Gross domestic product*	−0.06	<0.001	−0.04	0.409	0.07	0.006	0.10	<0.001	−0.11	0.01	−0.25	<0.001
*Education index*	−0.38	<0.001	−0.83	<0.001	−0.32	<0.001	−0.19	<0.001	−0.26	<0.001	−0.90	<0.001
*Unemployment*	−0.03	0.002	0.001	0.979	0.02	0.116	−0.034	<0.001	0.02	0.363	0.01	0.224
*Injuries prevalence*	−0.11	<0.001	−0.13	0.040	0.21	<0.001	0.10	<0.001	0.65	<0.001	0.42	<0.001
*Urbanization*	−0.07	<0.001	−0.10	0.005	−0.34	<0.001	0.06	0.047	0.15	<0.001	0.47	<0.001
*Air pollution*	0.14	<0.001	−0.04	0.258	0.15	<0.001	0.07	<0.001	0.29	<0.001	−0.10	<0.001
*Basic drinking-water services*	−0.20	<0.001	−0.60	<0.001	0.31	<0.001	−0.34	<0.001	−0.19	0.03	0.08	0.112
*Alcohol use*	0.05	<0.001	0.20	<0.001	0.07	<0.001	0.05	<0.001	−0.04	0.153	−0.07	<0.001
*Drug use*	−0.01	0.598	0.05	0.036	0.02	0.142	0.01	0.393	−0.41	<0.001	−0.26	<0.001
*Smoking*	0.03	0.073	−0.03	0.274	−0.22	<0.001	−0.16	<0.001	−0.15	<0.001	0.28	<0.001
*Prevalence of obesity*	0.40	<0.001	0.21	0.002	0.02	0.553	0.06	0.079	0.92	<0.001	0.10	<0.001
*Sexually transmitted infections*	0.75	<0.001	0.16	0.032	0.01	0.828	0.37	<0.001	−0.08	0.161	0.26	<0.001
*Year*	−0.03	<0.001	−0.002	0.565	−0.01	<0.001	−0.001	0.317	−0.06	<0.001	0.01	<0.001
YLL												
*Current health expenditure*	−0.04	0.012	−0.13	0.017	−0.18	<0.001	−0.36	<0.001	−0.05	0.210	−0.35	<0.001
*Gross domestic product*	−0.06	0.001	−0.02	0.751	0.13	0.001	0.18	<0.001	−0.14	0.019	−0.31	<0.001
*Education index*	−0.45	<0.001	−0.90	<0.001	−0.46	<0.001	−0.29	0.001	−0.35	<0.001	−0.88	<0.001
*Unemployment*	−0.03	0.001	−0.01	0.648	0.03	0.09	−0.05	<0.001	0.04	0.086	0.01	0.515
*Injuries prevalence*	−0.14	<0.001	−0.13	0.131	0.29	<0.001	0.19	<0.001	0.92	<0.001	0.56	<0.001
*Urbanisation*	−0.09	<0.001	−0.10	0.047	−0.59	<0.001	0.004	0.933	0.17	<0.001	0.64	<0.001
*Air pollution*	0.23	<0.001	−0.10	0.066	0.15	0.015	0.12	<0.001	0.48	<0.001	−0.10	<0.001
*Basic drinking-water services*	−0.24	<0.001	−0.70	0.003	0.49	<0.001	−0.35	0.006	−0.17	0.168	0.12	0.104
*Alcohol use*	0.06	<0.001	0.28	<0.001	0.11	<0.001	0.09	<0.001	−0.06	0.081	−0.17	<0.001
*Drug use*	−0.02	0.535	0.03	0.378	−0.002	0.937	−0.03	0.051	−0.56	<0.001	−0.34	<0.001
*Smoking*	0.03	0.075	−0.05	0.146	−0.33	<0.001	−0.21	<0.001	−0.14	0.017	0.50	<0.001
*Prevalence of obesity*	0.48	<0.001	0.26	0.005	0.04	0.37	0.17	0.001	0.94	<0.001	0.12	<0.001
*Sexually transmitted infections*	0.90	<0.001	0.31	0.003	0.06	0.424	0.68	<0.001	−0.04	0.579	0.25	<0.001
*Year*	−0.03	<0.001	−0.002	0.610	−0.01	<0.001	−0.003	0.827	−0.08	<0.001	0.02	<0.001
YLD												
*Current health expenditure*	−0.01	0.001	0.03	<0.001	−0.02	<0.001	0.01	<0.001	0.04	<0.001	−0.04	<0.001
*Education index*	−0.03	<0.001	−0.13	<0.001	0.02	0.161	0.003	0.827	−0.03	0.014	−0.05	0.015
*Injuries prevalence*	0.07	<0.001	0.01	0.295	0.08	<0.001	−0.01	0.172	0.24	<0.001	0.12	<0.001
*Urbanisation*	0.02	<0.001	−0.08	<0.001	−0.02	0.006	−0.01	0.120	0.05	<0.001	0.02	0.011
*Air pollution*	0.01	0.266	0.04	<0.001	0.07	<0.001	−0.003	0.275	0.10	<0.001	−0.04	<0.001
*Basic drinking-water services*	−0.02	0.029	−0.16	<0.001	−0.08	<0.001	−0.000	0.987	−0.09	<0.001	−0.09	<0.001
*Drug use*	0.04	<0.001	0.09	<0.001	0.06	<0.001	0.04	<0.001	−0.09	<0.001	0.04	<0.001
*Prevalence of obesity*	0.01	0.228	0.09	<0.001	0.01	0.075	0.01	0.221	0.14	<0.001	0.02	<0.001
*Sexually transmitted infections*	0.18	<0.001	0.05	<0.001	−0.01	0.236	−0.05	<0.001	−0.18	<0.001	0.14	<0.001
*Year*	−0.002	<0.001	−0.001	<0.001	0.000	0.144	−0.003	<0.001	−0.01	<0.001	0.001	0.004

*β* – regression coefficient, DALYs – disability-adjusted life years, WHO – World Health Organization, YLD – years lived with disability, YLL – years of life lost

We found the biggest negative impact of urbanisation on DALYs in the Eastern Mediterranean region (*β* = −0.34, *P* < 0.001) and the biggest positive impact in the Western Pacific region (*β* = 0.47; *P* < 0.001); however, according to the global findings (**Table 1**), there is a negative relationship between urbanisation and DALY (*β* = −0.21; *P* < 0.001). The effect of the unemployment variable on increasing the DALY indicator was not significant in any region (**Table 2**).

The regional analyses for the YLL indicator were almost similar to the DALY findings, while the YLD indicator findings differed. Here, the variables of sexually transmitted infection in the African region; education, drinking water, alcohol use, and drug use in the Americas region; urbanisation in the Eastern Mediterranean region; current health expenditure and unemployment in Europe; injuries, air pollution, and obesity in South-East Asia region; and gross domestic product and smoking in the Western Pacific region had the highest effect size with YLL indicator. While the analysis for the YLD indicator showed that the variables of sexually transmitted infection in the African region; education, urbanisation, drinking water, and drug use in the Americas region; injuries, air pollution and obesity in the South-East Asia region; and current health expenditure in the Western Pacific region had the highest effect size with the YLD indicator (*P* < 0.05) (**Table 2**).

Regarding other regional findings, we found that the highest *β *coefficients, representing the influence of the year on the change in the DALY indicator, in South-East Asia (*β* = −0.06; *P* < 0.001) and Africa (*β* = −0.03; *P* < 0.001), respectively. Additionally, with the exception of the drug use variable, the impact of the other determinant variables on explaining YLL was greater than that on YLD (*P* < 0.05) (**Table 2**).

### Visualising data through graphs

**Figure 1:**
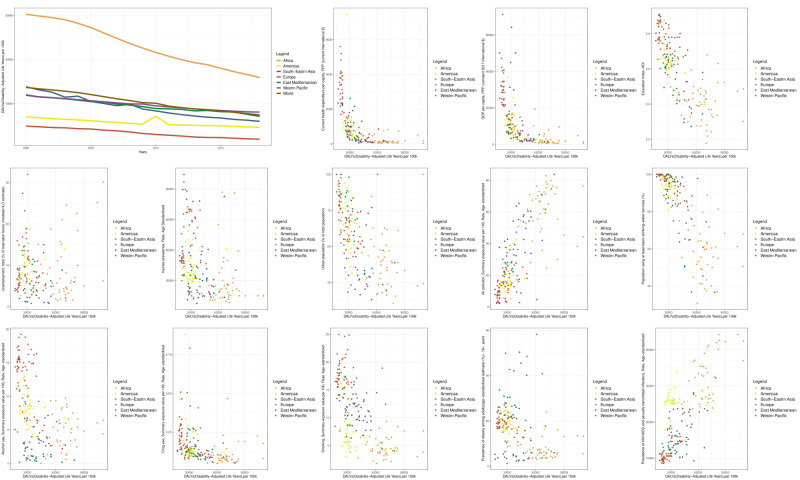
Time trend diagram of the DALY indicator and scatter diagrams of DALY determinants worldwide and in WHO regions. Each data point on scatter diagrams represents the average value of the respective indicators between 2000 and 2018 for a given country. Notably, all indicators for which scatter diagrams were generated, following both univariate and multiple analyses at the global level, demonstrated a significant association with the DALY indicator.

Regarding changes in the time trend of the DALY indicator between 2000 and 2018, we observed that the regions of Europe, the Americas, Eastern Mediterranean, Western Pacific, Southeast Asia, and Africa had the lowest DALY indicator in 2000, meaning the best health status. Meanwhile, the regions of Europe, the Americas, Southeast Asia, the Eastern Mediterranean, the Western Pacific, and Africa had the lowest DALY indicators in 2018 (arranged in ascending order of DALY indicators). This suggest that the health status in Southeast Asia has improved during this period. When comparing the status of the DALY indicator worldwide and across regions, it becomes evident that in 2000, Africa and Southeast Asia, and in 2018, Africa and the Western Pacific, had more unfavourable circumstances compared to the global average (**Figure 1**).

**Figure 2:**
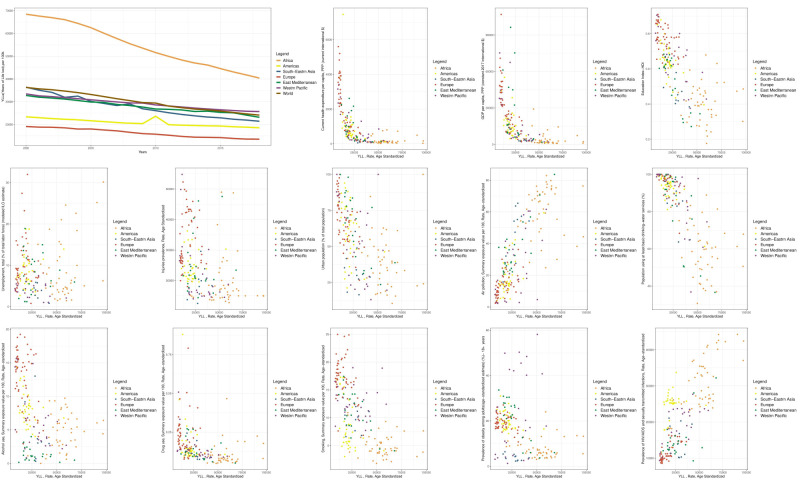
Time trend diagram of the YLL indicator and scatter diagrams of YLL determinants worldwide and in WHO regions. Each data point on scatter diagrams represents the average value of the respective indicators between 2000 and 2018 for a given country. Notably, all indicators for which scatter diagrams were generated, following both univariate and multiple analyses at the global level, demonstrated a significant association with the YLL indicator.

According to changes in the time trend of the YLL indicator, we observed the lowest YLL indicator in 2000 for Europe, the Americas, Eastern Mediterranean, the Western Pacific, Southeast Asia, and Africa (arranged in ascending order of YLL indicators) (**Figure 2**). In that year, the global average YLL indicator was higher than in the regions of Europe, the Americas, the Eastern Mediterranean, and the Western Pacific, but lower than in the regions of Southeasts Asia and Africa. In 2018, Europe, the Americas, South-East Asia, Eastern Mediterranean, Western Pacific, and Africa had the lowest level of this indicator (arranged in ascending order of YLL indicators). A comparison of the time trend changes of the YLL indicator in the WHO areas between these two periods shows that the position of the Southeast Asian region has improved.

**Figure 3:**
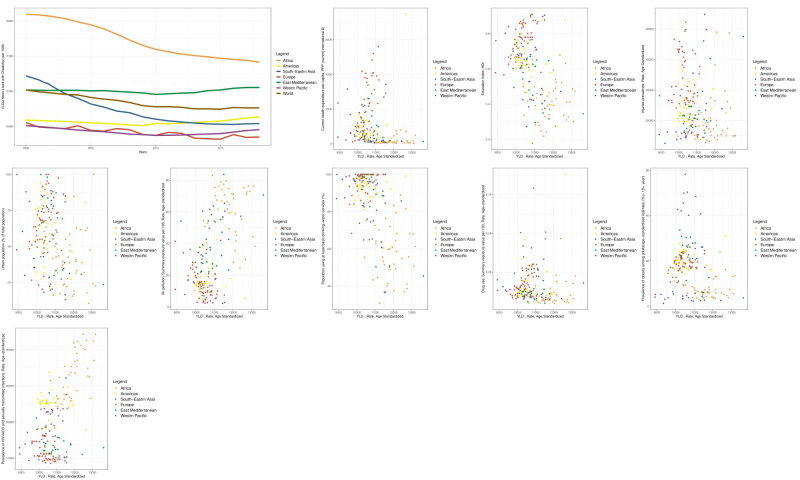
Time trend diagram of the YLD indicator and scatter diagrams of YLD determinants worldwide and in WHO regions. Each data point on scatter diagrams represents the average value of the respective indicators between 2000 and 2018 for a given country. Notably, all indicators for which scatter diagrams were generated, following both univariate and multiple analyses at the global level, demonstrated a significant association with the YLD indicator.

Additionally, in view of changes in the time trend of the YLD indicator between the 2000 and 2018, we found that the Western Pacific, Europe, the Americas, Eastern Mediterranean, Southeast Asia, and Africa had the lowest YLD indicators in 2000 (arranged in ascending order of YLD indicators). Meanwhile, the regions of Europe, the Western Pacific, Southeast Asia, the Americas, the Eastern Mediterranean, and Africa had the lowest levels of this indicator in 2018. Unlike the DALY and YLL indices, the YLD indicator during this period usually exhibited an irregular trend, with only Southeast Asia and Africa showing a constant and significant decreasing trend. According to the YLD indicator, Southeast Asia had a worse situation than the global average in 2000, yet the circumstances in 2018 than the global average (**Figure 3**).

## DISCUSSION

Our global-level findings showed that the variables of injuries prevalence, sexually transmitted infections, and urbanisation played a stronger role in explaining the DALY indicator. Meanwhile, all variables had a greater impact in explaining the YLD than the YLL indicator except for the drug use variable, as shown in regional findings.

According to our results, the variable of current health expenditure had the greatest impact on the expression of DALY and YLL indices in the European region and the lowest in the African region. Notably, the variable of sexually transmitted infections in the African region had the highest influence in explaining the DALY, YLL, and YLD indices compared to other regions. Hence, in the context of interregional policies, it is likely that investing in current health expenditure in the European region and addressing sexually transmitted infections in the African region will prove to be more efficient and effective than in other regions. Previous studies have shown that the highest-risk sexual behaviours often result in sexually transmitted infections and unintended pregnancies, particularly among economically disadvantaged populations [16,17]. Therefore, it is logical to observe a higher association between the variable of sexually transmitted infections and health indicators in the African region compared to other regions.

Based on the DALY indicator, the variables of drinking water, alcohol use, and drug use appear to have the most significant impact on health outcome in the Americas region compared to other WHO regions. Similarly, in terms of the YLL indicator, the variables of education, drinking water, alcohol use, and drug use exhibit the most substantial influence. Additionally, when considering the YLD indicator, the variables of education, urbanisation, drinking water, and drug use are more influential in the Americas region. Therefore, given that these determinants likely have a higher marginal impact on health in this region compared to other regions, it is imperative to prioritise them in interregional health policies. A previous study reported that the human population is growing faster than the amount of accessible freshwater and that per capita access to freshwater will decrease in the next century; climate change will cause a general intensification of the earth's hydrological cycle in the next 100 years – precipitation, evapotranspiration, and the occurrence of storms will increase, and significant changes in biogeochemical processes will affect water quality [18]. Regarding alcohol, a previous study conducted across several continents and selected countries showed that alcohol consumption is higher among the people of the Americas, Europe, Japan, and New Zealand, with a lower proportion in the Middle East, Africa, and China [19]. Another study found that individuals experiencing socio-economic deprivation are at an increased risk of harmful drug use. This risk is also elevated for those in disadvantaged family environments and those residing in communities with higher levels of substance use. Substance use is increasing in low-income countries, which are expected to bear a disproportionate burden of substance-related disability and premature death in the coming decades [20]. Therefore, there is a definitive need to pay attention to these variables as determinants of health.

According to our findings, the urbanisation variable had the most significant impact on reducing DALY and YLL in the Eastern Mediterranean region and increasing them in the Western Pacific region. A previous study showed that poverty is rapidly shifting from rural to urban areas; in relation to this, food insecurity and malnutrition in all its forms are highly prevalent among urban dwellers, and the rapid increase in overweight and obesity in urban areas is a concern [21].

We also observed that the role of variables of injuries, air pollution, and obesity in explaining the DALY, YLL, and YLD indices was more pronounced in Southeast Asia compared to other regions. It seems that prioritising the aforementioned variables in this region would be more cost-effective than in doing so elsewhere, considering that they would see the most significant change in health indicators with every percentage change in said variables. In a study conducted in 2017, among unintentional injury deaths, drowning was the leading cause of death for children aged 1–4 years; motor vehicle accidents were the leading cause of death for those aged 5–24 years; and unintentional poisoning was the leading cause of death for people aged 25–64 [22]. In a previous study, the effects of PM_10_ and CO_2_ pollutants on infant mortality and life expectancy in 60 developing countries during 1990–2010 showed that the benefits of improved health status through improved socioeconomic conditions can be cancelled out by PM_10_ and CO_2_ air pollutants. Therefore, health policies that focus only on socioeconomic aspects and ignore the adverse effects of air pollution may do little in efforts directed towards improving the current health status of developing countries [23]. Another study that analysed health survey data from Australia, Canada, England, and Korea showed that better education is associated with a lower likelihood of obesity, especially among women [24], while another found the highest average of all indicators of subcutaneous fat, general obesity, and central obesity in the lowest educational group and the least average in the highest educational group [25].

Regarding the DALY indicator, the variables of gross domestic product, education, and smoking appear to have the most substantial impact on health status in the Western Pacific region compared to other regions. Similarly, concerning the YLL indicator, the variables of gross domestic product and smoking exhibit the greatest influence. Additionally, when considering the YLD indicator, the current health expenditure variable shows a stronger impact in the Western Pacific region. Therefore, if interregional health policies are to be adopted to promote global health, it is imperative to give special attention to these variables in the Western Pacific region. This is because the marginal impact of these variables is likely higher in the Western Pacific region compared to other regions. According to previous studies, tobacco smoking is the leading cause of death from non-communicable diseases and the second leading risk factor for early death and disability worldwide [26,27].

### Limitations

One of the limitations of our study was the incompleteness of the data related to some variables, including family and social support. Consequently, as outlined in the study's methodology, we excluded indicators or variables with <80% data coverage from the analysis. The second limitation stems from the fact that the most recent data available from the GBD is from 2019. Consequently, we had to use data from 2000 to 2018. Despite significant global changes in recent years due to the coronavirus disease 2019 (COVID-19) pandemic, this data set does not cover that time period. The third limitation is associated with the difficulty of comparing various variables within a specific region because of the diverse nature of the indicators. Therefore, in our interpretation of the findings, we tried to compare individual variables across regions rather than different variables within a single region. The fourth limitation of our study is related to the ecological fallacy inherent to ecological studies, which involves assuming that relationships between variables at the overall level remain consistent at the individual level [28]. To mitigate this limitation, we exercised caution when interpreting the results and making inferences at both the global and regional levels, avoiding direct attribution to individual countries or individuals.

## CONCLUSIONS

Our results, which are based on global evidence, can be useful in reducing health inequalities and gaps in all regions, especially in those where determinants play a more significant role. To achieve greater health equality and efficient allocation of resources, our study should be considered in producing and implementing global and regional policies.

## Additional material


Online Supplementary Document
