# Supplementary material for: Assessing health determinants worldwide: Econometric analysis of the Global Burden of Diseases Study 2000–18 – Highlighting impactful factors on DALY, YLL, and YLD indicators
Source: J Glob Health. 2024 Mar 15;14:04051. doi: 10.7189/jogh.14.04051 (PMC10939113; doi:10.7189/jogh.14.04051)
Supplement: Online Supplementary Document [file jogh-14-04051-s001.pdf]

ONLINE SUPPLEMENTARY DOCUMENT

Title: Assessing Health Determinants Worldwide: Econometric Analysis of the Global Burden of Diseases Study 2000-2018, Highlighting Impactful Factors on DALY, YLL and YLD Indicators

Authors: Kamran Irandoust[1,2*], Rajabali Daroudi[1*], Maryam Tajvar[1†], Mehdi Yaseri[3†]

[1] Department of Health Management, Policy, and Economics, School of Public Health, Tehran University of Medical Sciences, Tehran, Iran

[2] Department of Health Economics, School of Health Management and Information Sciences, Iran University of Medical Sciences, Tehran, Iran

[3] Department of Epidemiology and Biostatistics, School of Public Health, Tehran University of Medical Sciences, Tehran, Iran

[*]Joint first authorship.

[†]Joint senior authorship.

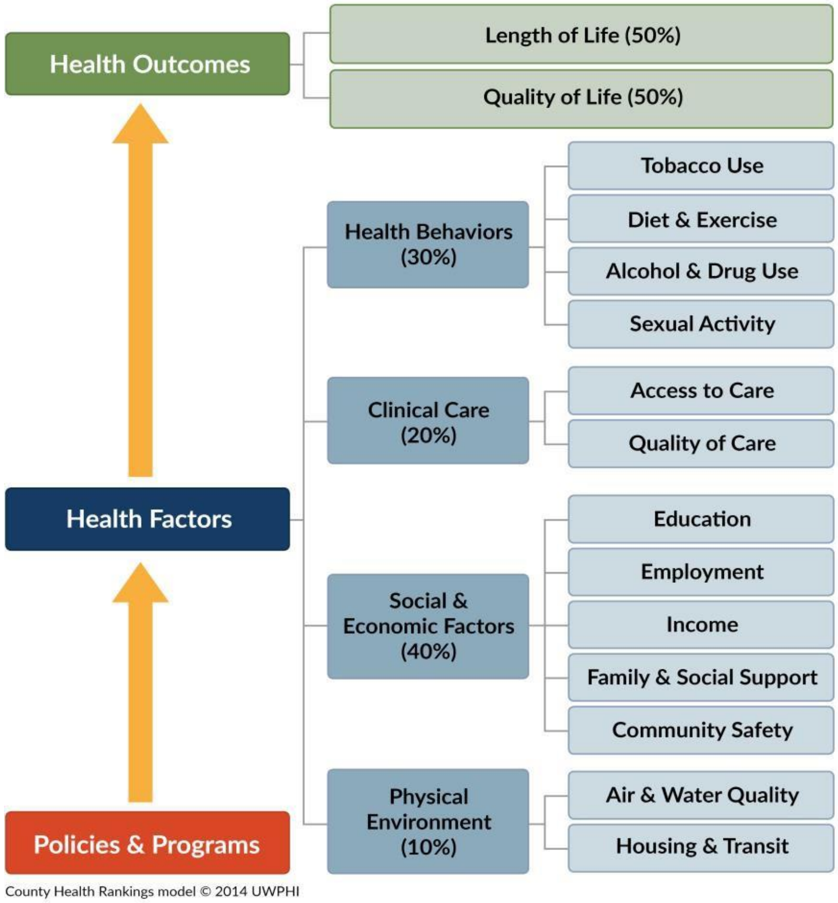

Figure S1. County Health Rankings & Roadmaps (CHR&R) Model

Source: County Health Rankings model © 2014, University of Wisconsin Population Health Institute.

Available: https://www.countyhealthrankings.org/explore-health-rankings/county-health-rankings-model

Table S1. dimensions and indicators of measuring health status

Dimension	Indicators	Description	%DA
**Health Outcomes**	Years of Life Lost (YLL)	Rate, Age Standardized	100.00
	Years Lived with Disability (YLD)	Rate, Age Standardized	100.00
	Disability-Adjusted Life-Years (DALYs)	Rate, Age Standardized	100.00

DA – data availability

Table S2. dimensions, components, and indicators of measuring health determinants

Dimension	Component	Indicators	Description	%DA
**Clinical Care**	Access to Care	Current health expenditure	per capita, PPP (current international $)	90.31
	Quality to Care	Domestic general government health expenditure	per capita, PPP (current international $)	90.80
**Social & Economic Factors**	Income	Gross domestic product (GDP)	per capita, PPP (constant 2017 international $)	93.24
		Income Index †	Income Index-HDI	97.21
		Income inequality *	GINI index (World Bank estimate)	33.97
	Education	Years of schooling †	Mean years of schooling (years)	94.38
		Literacy *	rate, adult total (% of people ages 15 and above)	18.50
		Education Index	Education Index-HDI	94.00
	Employment	Unemployment	% Of total labor force (modeled ILO estimate)	82.09
	Community Safety	Injuries prevalence	Rate, Age Standardized	100.00
		Intentional homicides *	Intentional homicides (per 100,000 people)	62.56
	Family & Social Support	Social protection *	CPIA social protection rating (1=low to 6=high)	28.51
		Coverage of social safety net program *	% Population- Coverage (%) - All Social Assistance	10.15
**Physical Environment**	Housing & Transit	Urban population (Urbanization)	Urban population (% of the total population)	98.78
		Population density †	Population density (people per sq. km of land area)	97.75
		Poverty *	Poverty gap at $3.20 a day (2011 PPP) (%)	34.16
	Air & Water Quality	PM2.5 air pollution *	mean annual exposure (micrograms per cubic meter)	50.46
		CO2 emissions *	CO2 emissions (metric tons per capita)	74.58
		Air pollution	Summary exposure value per 100, Rate, Age-standardiz	100.00
		Basic drinking-water services	Population using at least (%)	92.11
		Managed drinking water services *	People using safely (% of the population)	46.85
		Unsafe water, sanitation, hand washing †	Summary exposure value per 100, Rate, Age-	100.00
**Health Behaviors**	Alcohol & Drug Use	Alcohol consumption †	recorded per capita (15+) (in liters of pure alcohol)	94.93
		Prevalence of Alcohol use disorders †	Rate, Age-standardized	99.86
		Alcohol use	Summary exposure value per 100, Rate, Age-standardized	100.00
		Prevalence of Drug use disorders †	Rate, Age-standardized	99.32
		Drug use	Summary exposure value per 100, Rate, Age-standardized	99.92
	Tobacco Use	Smoking prevalence *	Smoking prevalence, total (ages 15+)	33.45
		Tobacco †	Summary exposure value per 100, Rate, Age-standardized	98.45
		Smoking	Summary exposure value per 100, Rate, Age-standardized	100.00
	Diet & Exercise	Oil consumption *	per capita (tones per year per person)	28.13
		Consumption of iodized salt *	Consumption of iodized salt (% of households)	8.11
		Sugar consumption *	Sugar per person (g per day)	57.03
		Prevalence of obesity among adults	BMI & Greater Equal, 30 (age-standardized) (%)	87.17
		Prevalence of overweight †	Prevalence of overweight (% of adults)	86.25
	Sexual Activity	HIV & sexually transmitted infections	Prevalence, Rate, Age-standardized	99.48

DA – data availability

* Indicators where the amount of data availability was less than 80%.

† Indices that overlapped.

Table S3. World Health Organization member countries by region- 2020

WHO Region	194 Member countries
**Africa (47 countries)**	Algeria, Angola, Benin, Botswana, Burkina Faso, Burundi, Cabo Verde, Cameroon, Central African Republic, Chad, Comoros, Congo, Cote d'Ivoire, Democratic Republic of Congo, Equatorial Guinea, Eritrea, Eswatini, Ethiopia, Gabon, Gambia, Ghana, Guinea, Guinea-Bissau, Kenya, Lesotho, Liberia, Madagascar, Malawi, Mali, Mauritania, Mauritius, Mozambique, Namibia, Niger, Nigeria, Rwanda, Sao Tome and Principe, Senegal, Seychelles, Sierra Leone, South Africa, South Sudan, Togo, Uganda, United Republic of Tanzania, Zambia, Zimbabwe
**Americas (35 countries)**	Antigua and Barbuda, Argentina, Bahamas, Barbados, Belize, Bolivia, Brazil, Canada, Chile, Colombia, Costa Rica, Cuba, Dominica, Dominican Republic, Ecuador, El Salvador, Grenada, Guatemala, Guyana, Haiti, Honduras, Jamaica, Mexico, Nicaragua, Panama, Paraguay, Peru, Saint Kitts and Nevis, Saint Lucia, Saint Vincent and the Grenadines, Suriname, Trinidad and Tobago, United States of America, Uruguay, Venezuela (Bolivarian Republic of)
**South-East Asia (11 countries)**	Bangladesh, Bhutan, Democratic People's Republic of Korea, India, Indonesia, Maldives, Myanmar, Nepal, Sri Lanka, Thailand, Timor-Leste
**Europe (53 countries)**	Albania, Andorra, Armenia, Austria, Azerbaijan, Belarus, Belgium, Bosnia and Herzegovina, Bulgaria, Croatia, Cyprus, Czechia, Denmark, Estonia, Finland, France, Georgia, Germany, Greece, Hungary, Iceland, Ireland, Israel, Italy, Kazakhstan, Kyrgyzstan, Latvia, Lithuania, Luxembourg, Malta, Monaco, Montenegro, Netherlands, North Macedonia, Norway, Poland, Portugal, Republic of Moldova, Romania, Russian Federation, San Marino, Serbia, Slovakia, Slovenia, Spain, Sweden, Switzerland, Tajikistan, Turkey, Turkmenistan, Ukraine, United Kingdom of Great Britain and Northern Ireland, Uzbekistan
**Eastern Mediterranean (21 countries)**	Afghanistan, Bahrain, Djibouti, Egypt, Iran Islamic Republic of, Iraq, Jordan, Kuwait, Lebanon, Libya, Morocco, Oman, Pakistan, Qatar, Saudi Arabia, Somalia, Sudan, Syrian Arab Republic, Tunisia, United Arab Emirates, Yemen
**Western Pacific (27 countries)**	Australia, Brunei Darussalam, Cambodia, China, Cook Islands, Fiji, Japan, Kiribati, Lao Peoples Democratic Republic, Malaysia, Marshall Islands, Micronesia (Federated States of), Mongolia, Nauru, New Zealand, Niue, Palau, Papua New Guinea, Philippines, Republic of Korea, Samoa, Singapore, Solomon Islands, Tonga, Tuvalu, Vanuatu, Viet Nam

Table S4. List of databases used in the study and their access link

Database	Database URL
World Bank	https://databank.worldbank.org/source/world-development-indicators
World Health Observatory	https://apps.who.int/gho/data/node.imr
Global Health Expenditure Database	https://apps.who.int/nha/database/Select/Indicators/en
Gapminder	https://www.gapminder.org/data/
United Nations Human Development Reports	http://hdr.undp.org/en/data
Global Burden of Disease Studies	http://ghdx.healthdata.org/gbd-results-tool

Table S5. The results of the univariate test of the multilevel mixed-effects linear regression model

	Indicators	RC	CI (95%)	*P*-value
**DALY**	Current health expenditure	4·05	3·55 to 4·55	<0·001
	Domestic general government health expenditure	5·29	4·61 to 5·96	<0·001
	Gross domestic product (GDP)	0·09	0·03 to 0·15	0·004
	Income Index	-58262·86	-64847·41 to -51678·31	<0·001
	Income inequality *	-41·86	-126·01 to 42·30	0·330
	Years of schooling	-833·65	-1275·03 to -392·28	<0·001
	Literacy *	-411·43	-493·96 to -328·90	<0·001
	Education Index	-53094·69	-59807·64 to - 46381·74	<0·001
	Unemployment	202·39	113·69 to 291·09	<0·001
	Injury's prevalence	0·45	0·34 to 0·56	<0·001
	Intentional homicides *	104·28	59·85 to 148·71	<0·001
	Social protection *	-3623·17	-4998·23 to -2248·12	<0·001
	Coverage of social safety net program *	-54·34	-94·71 to -13·96	0·008
	Urban population (Urbanization)	-494·17	-559·68 to -428·66	<0·001
	Population density	-6·80	-11·83 to -1·78	0·008
	Poverty *	367·49	306·19 to 428·78	<0·001
	PM2·5 air pollution *	-23·80	-108·32 to 60·72	0·581
	CO2 emissions *	-199·09	-319·58 to -78·61	0·001
	Air pollution	705·34	651·074 to 759·60	<0·001
	Basic drinking-water services	-563·05	-611·62 to -514·49	<0·001
	Managed drinking water services *	-112·52	-146·02 to -79·01	<0·001
	Unsafe water, sanitation, hand washing	424·94	377·30 to 472·59	<0·001
	Alcohol consumption	-631·79	-829·95 to -433·63	<0·001
	Prevalence of Alcohol use disorders	1·49	-0·04 to 3·02	0·057
	Alcohol use	-237·39	-401·23 to -73·54	0·005
	Prevalence of Drug use disorders	4·00	1·90 to 6·09	<0·001
	Drug use	23313·17	16579·52 to 30046·83	<0·001
	Smoking prevalence *	7·50	-98·05 to 113·04	0·889
	Tobacco	-983·46	-1122·96 to -843·96	<0·001
	Smoking	-1905·37	-2128·69 to -1682·04	<0·001
	Oil consumption *	692·12	-50·50 to 1434·75	0·068
	Consumption of iodized salt *	26·35	-46·71 to 99·41	0·480
	Sugar consumption *	-3·92	-29·42 to 21·59	0·763
	Prevalence of obesity among adults	1925·34	1724·21 to 2126·46	<0·001
	Prevalence of overweight	-383·68	-514·78 to -252·58	<0·001
	HIV & sexually transmitted infections	2·30	2·13 to 2·47	<0·001
**YLL**	Current health expenditure	3·92	3·43 to 4·41	<0·001
	Domestic general government health expenditure	5·12	4·46 to 5·78	<0·001
	Gross domestic product (GDP)	0·09	0·03 to 0·14	0·003
	Income Index	-56444·26	-628554·26 to -50034·26	<0·001
	Income inequality *	-44·19	-126·72 to 38·35	0·294
	Years of schooling	-822·52	-1252·78 to -392·27	<0·001
	Literacy *	-389·70	-469·82 to -309·58	<0·001
	Education Index	-51319·6	-57862·09 to -44777·1	<0·001
	Unemployment	199·63	111·93 to 287·34	<0·001
	Injury's prevalence	0·39	0·27 to 0·50	<0·001
	Intentional homicides *	101·51	56·71 to 146·30	<0·001
	Social protection *	-3528·63	-4879·83 to -2177·421	<0·001
	Coverage of social safety net program *	-48·82	-88·17 to -9·48	0·015

	Urban population (Urbanization)	-468·57	-534·33 to -402·81	<0·001
	Population density	-6·40	-11·30 to -1·49	0·011
	Poverty *	352·93	292·14 to 413·72	<0·001
	PM2·5 air pollution *	-20·17	-102·72 to 62·38	0·632
	CO2 emissions *	-80·12	-317·98 to -80·12	0·001
	Air pollution	677·71	624·87 to 730·54	<0·001
	Basic drinking-water services	-593·85	-593·85 to -495·97	<0·001
	Managed drinking water services *	-108·53	-141·28 to -75·78	<0·001
	Unsafe water, sanitation, hand washing	416·45	370·44 to 462·45	<0·001
	Alcohol consumption	-615·73	-809·36 to -422·12	<0·001
	Prevalence of Alcohol use disorders	1·15	-0·36 to 2·66	0·137
	Alcohol use	-233·26	-393·27 to -73·24	0·004
	Prevalence of Drug use disorders	4·47	2·39 to 6·54	<0·001
	Drug use	22313·68	15734·08 to 28893·28	<0·001
	Smoking prevalence *	-7·19	-110·98 to 96·59	0·892
	Tobacco	-989·26	-1125·82 to -852·70	<0·001
	Smoking	-1831·42	-2049·78 to -1613·06	<0·001
	Oil consumption *	726·66	-3·196 to 1459·52	0·051
	Consumption of iodized salt *	24·90	-46·12 to 95·90	0·492
	Sugar consumption *	-5·24	-30·79 to 20·30	0·687
	Prevalence of obesity among adults	1765·17	1572·38 to 1957·95	<0·001
	Prevalence of overweight	-382·09	-509·22 to -254·95	<0·001
	HIV & sexually transmitted infections	2·20	2·04 to 2·37	<0·001
**YLD**	Current health expenditure	0·12	0·10 to 0·14	<0·001
	Domestic general government health expenditure	0·15	0·12 to 0·18	<0·001
	Gross domestic product (GDP)	0·001	-0·001 to 0·004	0·240
	Income Index	-1855·03	-2139·18 to -1570·88	<0·001
	Income inequality *	2·92	-2·37 to 8·21	0·279
	Years of schooling	-30·50	-48·17 to -12·84	0·001
	Literacy *	-15·27	-18·86 to -11·69	<0·001
	Education Index	-1978·18	-2258·75 to -1697·61	<0·001
	Unemployment	7·02	3·53 to 10·51	<0·001
	Injury's prevalence	0·05	0·04 to 0·05	<0·001
	Intentional homicides *	4·18	2·46 to 5·91	<0·001
	Social protection *	-108·47	-160·96 to -55·99	<0·001
	Coverage of social safety net program *	-1·40	-2·76 to -0·03	0·045
	Urban population (Urbanization)	-14·51	-17·40 to -11·63	<0·001
	Population density	-0·46	-0·65 to -0·26	<0·001
	Poverty *	16·62	12·84 to 20·40	<0·001
	PM2·5 air pollution *	-0·29	-3·62 to 3·04	0·864
	CO2 emissions *	-6·38	-11·76 to -0·99	0·020
	Air pollution	26·33	23·83 to 28·83	<0·001
	Basic drinking-water services	-16·24	-18·23 to -14·25	<0·001
	Managed drinking water services *	-3·08	-5·12 to -1·04	0·003
	Unsafe water, sanitation, hand washing	2·56	-0·27 to 5·39	<0·076
	Alcohol consumption	-6·31	-14·11 to 1·48	<0·112
	Prevalence of Alcohol use disorders	0·16	0·10 to 0·23	<0·001
	Alcohol use	-5·37	-11·82 to 1·08	0·103
	Prevalence of Drug use disorders	0·42	0·33 to 0·50	<0·001
	Drug use	1256·80	994·40 to 1519·19	<0·001
	Smoking prevalence *	2·08	-2·89 to 7·06	0·412
	Tobacco	-27·26	-33·13 to -21·39	<0·001
	Smoking	-59·71	-69·06 to -50·36	<0·001

Oil consumption *	26·34	-10·45 to 63·14	0·161
Consumption of iodized salt *	1·13	-1·51 to 3·77	0·402
Sugar consumption *	0·34	-0·69 to 1·38	0·513
Prevalence of obesity among adults	103·48	95·88 to 111·07	<0·001
Prevalence of overweight	-1·35	-7·29 to 4·59	<0·001
HIV & sexually transmitted infections	0·09	0·08 to 0·10	<0·001

RC – regression coefficient, CI – confidence interval, DALYs – disability-adjusted life years, YLL – years of life lost, YLD – years lived with disability.
* Indicators where the amount of data availability was less than 80%.

Table S6. The final list of indicators that entered the econometric model

dependent variable	independent variables
**DALYs**	Current health expenditure; Gross domestic product (GDP); Education Index; Unemployment; Injuries prevalence; Urban population (Urbanization); Air pollution; Basic drinking-water services; Alcohol use; Drug use; Smoking; Prevalence of obesity; Sexually transmitted infections
**YLL**	Current health expenditure; Gross domestic product (GDP); Education Index; Unemployment; Injuries prevalence; Urban population (Urbanization); Air pollution; Basic drinking-water services; Alcohol use; Drug use; Smoking; Prevalence of obesity; Sexually transmitted infections
**YLD**	Current health expenditure; Education Index; Injuries prevalence; Urban population (Urbanization); Air pollution; Basic drinking-water services; Drug use; Prevalence of obesity; Sexually transmitted infections

DALYs – disability-adjusted life years, YLL – years of life lost, YLD – years lived with disability.
